# Unraveling Heat Tolerance in Upland Cotton (*Gossypium hirsutum* L.) Using Univariate and Multivariate Analysis

**DOI:** 10.3389/fpls.2021.727835

**Published:** 2022-01-13

**Authors:** Muhammad Mubashar Zafar, Xue Jia, Amir Shakeel, Zareen Sarfraz, Abdul Manan, Ali Imran, Huijuan Mo, Arfan Ali, Yuan Youlu, Abdul Razzaq, Muhammad Shahid Iqbal, Maozhi Ren

**Affiliations:** ^1^Zhengzhou Research Base, State Key Laboratory of Cotton Biology, School of Agricultural Sciences, Zhengzhou University, Zhengzhou, China; ^2^Institute of Cotton Research, Chinese Academy of Agricultural Sciences, Anyang, China; ^3^Department of Plant Breeding and Genetics, University of Agriculture, Faisalabad, Pakistan; ^4^FB Genetics, Four Brothers Group, Lahore, Pakistan; ^5^Institute of Molecular Biology and Biotechnology, The University of Lahore, Lahore, Pakistan; ^6^Cotton Research Station, Ayub Agricultural Research Institute, Faisalabad, Pakistan

**Keywords:** high-temperature stress, upland cotton (*Gossypium hirsutum* L.), principal component analysis (PCA), heritability, *Gossypium*

## Abstract

The ever-changing global environment currently includes an increasing ambient temperature that can be a devastating stress for organisms. Plants, being sessile, are adversely affected by heat stress in their physiology, development, growth, and ultimately yield. Since little is known about the response of biochemical traits to high-temperature ambiance, we evaluated eight parental lines (five lines and three testers) and their 15 F_1_ hybrids under normal and high-temperature stress to assess the impact of these conditions over 2 consecutive years. The research was performed under a triplicate randomized complete block design including a split-plot arrangement. Data were recorded for agronomic, biochemical, and fiber quality traits. Mean values of agronomic traits were significantly reduced under heat stress conditions, while hydrogen peroxide, peroxidase, total soluble protein, superoxide dismutase, catalase (CAT), carotenoids, and fiber strength displayed higher mean values under heat stress conditions. Under both conditions, high genetic advance and high heritability were observed for seed cotton yield (SCY), CAT, micronaire value, plant height, and chlorophyll-a and b content, indicating that an additive type of gene action controls these traits under both the conditions. For more insights into variation, Pearson correlation analysis and principal component analysis (PCA) were performed. Significant positive associations were observed among agronomic, biochemical, and fiber quality-related traits. The multivariate analyses involving hierarchical clustering and PCA classified the 23 experimental genotypes into four groups under normal and high-temperature stress conditions. Under both conditions, the F_1_ hybrid genotype FB-SHAHEEN × JSQ WHITE GOLD followed by Ghuari-1, CCRI-24, Eagle-2 × FB-Falcon, Ghuari-1 × JSQ White Gold, and Eagle-2 exhibited better performance in response to high-temperature stress regarding the agronomic and fiber quality-related traits. The mentioned genotypes could be utilized in future cotton breeding programs to enhance heat tolerance and improve cotton yield and productivity through resistance to environmental stressors.

## Introduction

Cotton is the world’s most important natural fiber and oil crop (Patel et al., 2021; Salimath et al., 2021). Climate change in recent decades has resulted in outbreaks of biotic and abiotic stressors that negatively affect plant yield and quality. Among abiotic stressors, heat stress is one of the most detrimental constraints, limiting cotton production by disturbing its normal growth, physiological, and developmental processes. Pakistan ranks fifth among top cotton-producing countries after India, China, United States, and Brazil (Khan et al., 2020), even though the nations cotton zone average temperature ranks highest among cotton-growing areas worldwide (Saleem et al., 2021). In Pakistan, the average temperature of the cotton-growing belt remains at 37°C/25°C (day/night) as compared to the United States 30°C/24°C, China’s 29°C/18°C, and India’s 34°C/21°C (Saleem et al., 2021). During the early growth period (May–June), the temperature remains as high as 40–45°C, and at times reaches 50°C.

Like most crop plants, cotton is susceptible to heat stress, especially during the developmental (Zahid et al., 2016) and reproductive phases (Salman et al., 2019). The most notable effects include flower shedding at the flowering phase, leading to stunted growth and reduced boll weight, and ultimately lower yields (Xu et al., 2020). At the peak of the reproductive phase, exposure to heat stress very often results in reduced seed cotton yield (SCY); whereas slightly lower temperatures at this time are more favorable and produce a better yield (Sarwar et al., 2017). Previous studies report some of the optimum temperatures for various growth and developmental stages of the cotton crop: for cottonseed germination 12^°^C, root development 30^°^C, and seedling development for boll development 25.5–29.5°C (Conaty et al., 2012; Lokhande and Reddy, 2014). A temperature range of 32–40°C usually negatively impacts root development, and when the temperature rises to 36^°^C, stomatal conductance decreases. The weight of the boll reduces as the temperature rise from 25.5 to 29.5°C (Conaty et al., 2012; Lokhande and Reddy, 2014). Singh et al., 2007 reported that each 1°C rise of temperature in the field reduces the SCY by 110 kg ha^–1^. Pollen tube germination, growth, and elongation are adversely affected by a temperature increase from 28 to 30°C. At 28°C, optimum pollen germination occurs (Burke et al., 2004) and the germination rate decreases as the temperature rises sharply from 28 to 37°C. Therefore, high-temperature stress reduces the germination rate, plant growth, photosynthetic rate, fruiting branches, membrane integrity, boll weight, and increases boll abscission, all of which lead to lower yield (Salman et al., 2019). Heat stress is also related to reduced boll size and the number of seeds per boll that limit fertilization efficiency (Pettigrew, 2008). In Pakistan, the average boll weight is 2–3 g, which is lower than in other countries (Saleem et al., 2021). High-temperature stress decreases the chlorophyll content, ultimately reducing the photosynthetic rate and translocation of assimilates to reproductive organs and thus, increases senescence (Rafiq et al., 2013; Dabbert and Gore, 2014). High-temperature stress also distorts stomatal movement and stomatal conductance 28–30°C resulting in poor gaseous exchange, ultimately effecting photosynthesis and ultimately the productivity (Conaty et al., 2012).

This high temperature abiotic stress affects plant antioxidant activities, leading to decreased SCY (Kamal et al., 2017). Under high-temperature stress, oxidative stress is induced to generate reactive oxygen species (ROS) (Kocsy et al., 2004). Under heat stress, ROS such as hydrogen peroxide (H_2_O_2_), superoxide radical (O_2_^–^), singlet oxygen (^1^O_2_), and hydroxyl radicals are produced in higher amounts (Choudhury et al., 2013). Their increased production may irreversibly damage plant cells through the oxidation of different cellular compartments such as chloroplasts, peroxisomes, and mitochondria (Roychoudhury et al., 2012). In plants, tolerance to oxidative damage is directly correlated with the production of antioxidant enzymes (Almeselmani et al., 2009). To scavenge the damaging ROS, the cotton plant activates the production of detoxifying enzymes including superoxide dismutase (SOD), peroxidases (POD), catalase (CAT), and non-enzymatic antioxidants including carotenoids, flavonoids, ascorbate, and tocopherols (Suzuki et al., 2012). Since high-temperature stress is a detrimental factor in cotton production, the development of heat-tolerant germplasm is a prominent objective of today’s cotton breeders, who aim to achieve higher yields under heat stress (Teixeira et al., 2013).

Numerous approaches can be adopted to face and overcome high-temperature abiotic stress in cotton. Scientists consider the development of heat-tolerant germplasm as reliable, long-lasting, cost-effective, and the best possible solution for combating this stress. Through conventional breeding, a significant level of tolerance in cotton can be achieved. Moreover, the global mean temperature is constantly increasing, urging cotton breeders to search for hidden potential genotypes from the existing germplasm *via* effective screening approaches based on particular morphological, biochemical, and physiological traits (Salman et al., 2019). The selection of cotton genotypes tolerant to heat stress is a prerequisite for cotton breeding improvement programs.

Understanding multivariate statistics necessitates an understanding of high-dimensional geometry and a conceptualization of linear algebra (Stewart and Thomas, 2008). Unlike univariate and bivariate models, multivariate data addresses several issues simultaneously. Multivariate analysis can provide many options to test and summarize the power of linear relationships across multiple variables (Timm, 2002). For example, in correlation tests, parametric and non-parametric options are present while using this technique. Plant breeders can use multivariate analysis to understand differences across variables and their possible associations (Dhamayanthi et al., 2018). For using standard least-square fit, several reports state that environmental indicators may significantly correlate with quantitative traits, including crop yield (Zhang et al., 2010; Sellam and Poovammal, 2016; Zhou et al., 2021). Hence, simple ANOVA functions are usually inefficient for describing the effect of environmental indicators with the desired productivity and quality owing to higher complexity in a different set of variables (Hoaglin and Welsch, 1978; Goos and Meintrup, 2016). Principal component analysis (PCA) and cluster analysis have received more attention owing to impact during recent decades (Mohammadi and Prasanna, 2003). PCA can efficiently analyze the interaction among traits and the performance of genotypes and efficiently dissect trait association (Aslam et al., 2017). Cluster analysis groups have rows together that share similar values across several variables and are considered as an exploratory technique that helps to understand clumping structures in data (Rathinavel, 2018). Correlation analysis is mainly used to understand the degree of relationship and its nature across traits. It can deal with the basic notion of an association across various traits, which can help in the selection of genotypes with the desired combination of traits (Ghafoor et al., 2013).

Multivariate analyses comprise highly acceptable and precise methods and techniques to explore the potential genetic variation existing in the available germplasm (Malik et al., 2014). It can also exploit prospective genetic associations and patterns of variability within the studied germplasm. Worldwide, multivariate analyses are used to study a range of crops, especially maize, wheat, cotton, and sorghum (Ali et al., 2011; Ajmal et al., 2013; Jarwar et al., 2019). Hence, the current work was designed to evaluate suites of biochemical, morphological, and agronomic traits of available potential cotton existing genotypes *via* univariate and multivariate analyses under heat stress with the aim of developing new heat resilient cultivars. Such research would yield basic information regarding high-temperature resilience potential in existing genotypes that may prove valuable and advantageous for developing heat-tolerant cotton cultivars in cotton breeding improvement programs across heat-stricken climatic regions.

## Materials and Methods

### Plant Materials and Experimental Layout

During the normal cotton growing season of November 2017, 50 cotton genotypes were screened against heat stress based on SCY under field conditions at FB Genetics, Four Brothers Group, Pakistan. The experiment was performed in the field under two treatments, i.e., normal and heat stress (5–6°C above normal for 12 days at 50% flowering) following a split-plot arrangement under a randomized complete block design (RCBD) with two replications. The plant × plant and row × row distance was 30 and 75 cm, respectively, with a 6 m row length for each genotype under each replication. The temperature was raised by constructing a tunnel using polythene sheets for 12 days at 50% flowering and it was removed at night. After screening, eight cotton genotypes were selected as parents regarding their SCY under heat stress. The selected parental material was crossed in line × tester mating fashion in the following season to obtain the subsequent F_1_ hybrids. The five heat-tolerant genotypes were kept as female lines, namely, Ghuari-1, Badar-1, Eagle-2, CCRI-24, and Fb-Shaheen. The three sensitive genotypes were kept as male testers: Fb-Falcon, Fb-Smart 1, and JSQ White Gold.

The 15 F_1_ hybrids and their eight parents are listed in Table 1, and were planted at the field research area in the normal cotton growing season under normal and high-temperature stress conditions for 2 consecutive years, 2018 and 2019. Experimental layout was performed in a split-plot arrangement under RCBD. The plant × plant and row × row distance was kept as 30 and 75 cm, respectively, with a 6 m row length for each genotype under each replication. The seed of the selected genotypes was manually sown (dibble method) on furrows in June. The crop was harvested in October each year. The R × R and P × P distance was 75 and 30 cm, respectively. The experiment was laid out in a triplicated manner under RCBD following a split-plot arrangement. All the culture and agronomic practices were performed following local recommendations across crop-growing seasons over 2 years.

**TABLE 1:** List of lines, testers, and their cross combinations used in the experiment.

Lines	Testers	Crosses	Crosses	Crosses
Ghuari-1	Fb-Falcon	Ghuari-1 × Fb-Falcon	Badar-1 × Js White Gold	CCRI-24 × Fb-Smart1
Badar-1	Fb-Smart 1	Ghuari-1 × Fb-Smart1	Eagle-2 × Fb-Falcon	CCRI-24 × JSQ White Gold
Eagle-2	JSQ White Gold	Ghuari-1 × JSQ White Gold	Eagle-2 × Fb-Smart1	Fb-Shaheen × Fb-Falcon
CCRI-24		Badar-1 × Fb-Falcon	Eagle-2 × JSQ White Gold	Fb-Shaheen × Fb-Smart1
Fb-Shaheen		Badar-1 × Fb-Smart1	CCRI-24 × Fb-Falcon	Fb-Shaheen × JSQ White Gold

### Imposition of High-Temperature Stress

During September, when all genotypes were at the 50% flowering stage, heat stress was implemented for 12 days during both the years. The covering of the polythene tunnel enhanced the temperature (5–6°C) during the daytime and the tunnel was removed during the night (Muhammad et al., 2018). The minimum and maximum temperatures inside the tunnel were continuously recorded (Both et al., 2015) throughout the crop growing season (Supplementary Table 1A). After the implementation of high-temperature stress, data were collected regarding biochemical characters. The maximum and minimum temperature ranges recorded during the crop growing season are given in Supplementary Table 1B.

### Biochemical Traits

For the determination of biochemical traits, leaf samples were collected from the experimental genotypes after the imposition of high-temperature treatment for 12 days. The quantification of H_2_O_2_, CAT, peroxidase (POD), total soluble proteins (TSP), chlorophyll contents (Chl), and carotenoids (Car) in the leaves was performed to assess the effect of stress on biochemical attributes of the plants. For this purpose, the fourth fully expanded top leaf was considered for sampling from each genotype for biochemical analyses following the sampling method used by Song et al. (2014). Enzyme extraction was conducted on 0.5 g of cotton leaf samples. The leaves were cut with the help of a leaf pincher and then crushed and ground with 1–2 mL of chilled potassium phosphate buffer (pH 7.8). The prepared mixture was then centrifuged for 5 min at 1,400 rpm. Residues were discarded and the supernatant was collected for the determination of biochemical attributes *via* UV spectrophotometer at different wavelengths (Sarwar et al., 2019).

#### Hydrogen Peroxide (μmol/g-FW)

For the determination of H_2_O_2_, the Velikova protocol was followed (Velikova et al., 2000). Fresh leaf tissues (0.5 g) were blended by using trichloroacetic acid (TCA, 5 mL of 0.1% (w/v) solution) and then centrifuged at 12,000 rpm for 12 min. The supernatant was collected in a volume of 0.5 mL, and then 0.5 mL of phosphate buffer (pH 7.0) and 1 mL of potassium iodide were added. At the 390 nm wavelength of the UV spectrophotometer, the absorbance capacity of each sample was recorded.

#### Catalase (U/mg Protein)

Enzyme extract (0.1 mL) was mixed with 3 mL of the reaction mixture, containing 5.9 mM H_2_O_2_ and 50 mM potassium phosphate buffer (7.0 pH). CAT activity was recorded at the wavelength of 240 nm (Liu et al., 2009) using a spectrophotometer.

#### Peroxidase (U/mg Protein)

The POD solution contained 50 mM phosphate buffer (pH = 5), 40 mM H_2_O_2_, 20 mM guaiacol, and 0.1 mL of enzyme extract according to Liu’s protocol, after certain amendments (Liu et al., 2009). At 470 nm, absorbance changes were recorded by the spectrophotometer.

#### Total Soluble Proteins (mg/g-FW)

The Bradford reagent method was used for the determination of protein content. Aliquots of 100 μL of the sample were blended with 5 mL of Bradford reagent. At 595 nm wavelength, the absorbance was recorded (Bradford, 1976) using a spectrophotometer.

#### Chlorophyll Content and Carotenoids Assay

The Arnon method (Arnon, 1949) with specific alterations measured Chl a and b contents and carotenoid pigments. A volume of 8–10 mL of 80% acetone (v/v) was used for crushing a 0.50 g sample of the cotton leaf. Filter paper was used to obtain a homogenized solution. A spectrophotometer was employed to record the absorbance of the final solution at 645 and 663 nm wavelengths. Chl a and b contents and Car were estimated by using the following formulas.


$$\text{Chlorophyll}a\left(\frac{\mu \text{g}}{\text{g}}\text{FW}\right)=\left[12.7\left(\text{OD}663\right)-2.69\left(\text{OD}645\right)\right]\times \frac{\text{v}}{1000\times \text{w}}$$

Chlorophyllb (μggFW)=[22.9(OD665)-4.48(OD 663)]×V1000×w

$$\text{Carotenoids }\left(\frac{\mu \text{g}}{\text{g}}\text{FW}\right)=\frac{A^{\text{car}}}{\text{Em}}\times 1000$$

$$A^{car}=\text{OD}480+0.114\left(\text{OD}663\right)-0.638\left(\text{OD}645\right)$$

where,

*W* = weight of leaf sample, *V* = volume of sample, Em = 2,500

### Yield and Fiber Quality Traits

At crop agronomic maturity, data from five plants of each genotype regarding yield-related traits were recorded. The yield-related traits included plant height (PH), the number of bolls (TNB), boll weight (BW), SCY, and lint percentage (lint%). A representative sample from seed cotton obtained from the experimental genotypes was taken and weighed. The ginning of seed cotton samples was accomplished with the help of a single roller ginning machine (Testex, Model: TB510C) to separate seed and lint, and the ginning outturn was estimated by dividing the weight of lint in a sample by the seed cotton weight of the sample, expressed in percentage. Lint was further subjected to fiber quality analysis for the estimation of fiber strength (g/tex) (STR), short fiber (SF), micronaire value (MIC), reflectance (%) (RD), upper half mean length (mm) (UHML), and uniformity index ratio (%) (UI) with a high-volume instrument (HVI-900, USTER, United States), following ASTM protocol, publication D5867-05 for HVI analysis (ASTM, 2005).

### Statistical Analysis

The preliminary screening data comprising of 50 upland cotton accessions based on yield performance across heat and normal conditions were subjected to a linear mixed model using ANOVA, followed by the construction of an ANOM-decision chart to graphically represent the genotypic behavior for selection of parents for crossing through analysis of mean methods as described by Nelson et al. (2005). These analyses were performed using default and standardization options with SAS-JMP Pro 16 (SAS Institute Inc., Cary, NC, United States, 1989–2021). In this method, if a single or group of genotypes plotted statistics fell outside of the decision limits, then the test indicated a statistical difference between that group’s statistic and the overall average of the statistic for genotypes/groups (Yiğit and Mendeş, 2017). Based on the ANOM-decision charts, genotypes performing better across both normal and stress conditions having significant differences from means and falling above the upper decision level (UDL) can be considered as tolerant, whereas the genotypes having significant reduction of yield having a significant difference from means below the lower decision level (LDL) in stress treatment can be considered as susceptible.

Data collected from evaluation of parents and F_1_s across normal and heat stress for agronomic, biochemical, and fiber traits has been subjected to analysis of variance (Steel et al., 1997) to estimate genetic variability among parents and their subsequent hybrids. Means and standard errors were calculated and used throughout all the data sets (Gomez and Gomez, 1984). The years were then pooled to obtain mean values for further analyses. The method proposed by Singh and Chaudhary (1985) was used to calculate broad-sense heritability (H^2^b). For the categorization of H^2^b, a method based on a range of values was used, as follows: low H^2^b had a < 30% value, medium H^2^b values range between 30 and 70%, and high H^2^b values above 70%. The H^2^b was classified following the procedure given by Johnson (Johnson et al., 1955). Genetic advance percentage (GAM) is calculated by the method proposed by Poehlman and Sleper (1995) under a 20% selection intensity. For multivariate analyses, average data was taken for replications. Subsequently, means were subjected to multivariate analyses, including correlation matrix (Pearson correlation) and PCA (Correlation based), two-way cluster analysis (hierarchical clustering), and construction of a distance-based tree using Ward’s method; all these analyses were performed using default analyses and standardization options with SAS-JMP Pro 16 (SAS Institute Inc., Cary, NC, United States, 1989–2021).

To validate the results and also to determine the performance of genotypes with a high SCY and good fiber quality across normal and heat stress, 3D scatterplots were constructed based on stress tolerance indices, including mean performance (MP), geometric mean performance (GMP), and stress tolerance index (STI) for normal and heat stress with the help of the freely available online software package iPASTIC developed by Pour-Aboughadareh et al. (2019). To rank and identify the best genotypes having stable and better yields across both conditions, the representative trait was used according to the method given by Ketata et al. (1989). Based on this approach, the average sum of rank (ASR) corresponding to all variables/indices was used as an indicator for selecting the best genotypes. According to this procedure, the lowest rank was assigned to the genotype having the best performance for the corresponding variable; hence, genotypes with the lowest value for ASR and lowest values for standard deviation were denoted as the best ones.

## Results

**FIGURE 1:**
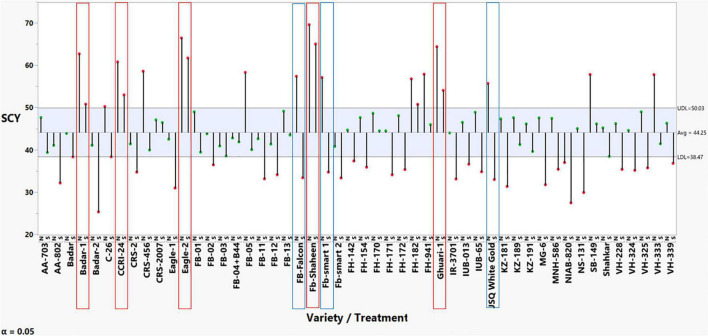
ANOM-decision chart with decision limits 38.47–50.03 for seed cotton yields across normal and heat stress (alpha > 0.05%). It provides a graphical test for simultaneously comparing the mean performance of these 50 cotton genotypes across normal and heat stress. Red-colored heads represent significant deviation from the mean, either above upper decision level (UDL) or below lower decision level (LDL).

The results from the preliminary screening experiment across normal and heat stress conditions, represented in Supplementary Table 2, revealed non-significant results for replications, whereas significant effect estimates were found for genotypes, treatment, and genotype treatment interaction for SCY. The results for mean comparisons through the analysis of mean methods (ANOM)-decision chart were constructed to represent the genotypic behavior for selection based on the ANOM. Based on the results shown in Figure 1, five genotypes were declared as tolerant since their means fell above the UDL across both normal and stress conditions without significantly decreasing SCY due to imposed stress. These five heat-tolerant cotton lines/genotypes were used as female lines for crossing: Ghuari-1, Badar-1, Eagle-2, CCRI-24, and Fb-Shaheen. We also selected three heat susceptible genotypes because they produced a significant decline in SCY across heat-stress conditions compared with a higher yield performance in normal conditions. These genotypes had a significantly higher yield in normal conditions, i.e., above the UDL, whereas they had significantly reduced yield performance in heat stress, i.e., below the LDL. These genotypes were: Fb-Falcon, Fb-Smart 1, and JSQ White Gold, and were selected to be used as testers (male parent) in crossing (Figure 1).

Analysis of variance showed significant differences among parents and F_1_ hybrids under heat stress during both years; this pointed toward the existence of genetic divergence (Table 2). The Genotypes × Treatment interaction for all traits was highly significant, suggesting that all parents and hybrids behaved differently under heat stress (Table 2). The analysis of variance for heat stress × year showed non-significant interactions for all traits except H_2_O_2_, POD, Chl a and b contents, STR, and MIC. The genotypes × year interaction showed non-significant interactions for all traits except SOD, TSP, Chl-a content, STR, MIC, and UI. The heat stress × year × genotypes interaction showed non-significant interactions for all traits except H_2_O_2_, SOD, TSP, Chl-b content, SF, STR, MIC, and UI (Table 2). Overall, heat stress negatively affected all agronomic and yield-related traits in all-cotton genotypes during both years. The following traits: PH, TNB, BW, SCY, Lint%, Chl a an b, SF, RD, and UI were reduced in all genotypes under high-temperature stress (Table 3). The mean values for H_2_O_2,_ TSP, STR, CAT, SOD, POD, and Car increased under heat stress whereas the mean values for Chl a and b decreased (Table 3).

**TABLE 2:** Summary of mean square values regarding the influence of high-temperature stress on different traits of the studied cotton genotypes of parents and their F_1_ hybrids across the 2 experimental years.

Traits		Genotypes		Heat Stress		Genotypes × Heat Stress		Heat stress × Year	Genotypes × Year	Heat stress × Year × genotype
		1st year	2nd year	1st year	2nd year	1st year	2nd year			
Plant Height		137.68**	151.7**	487.600	619.8	27.789**	30.0**	3.96	2.18	1.37
Number of bolls		36.650**	37.90**	877.93*	838.2*	14.092**	10.53**	0.23	1.61	1.26
Boll weight		0.2247**	0.184**	0.0088	0.182	0.0824**	0.04817	0.05	0.01	0.03
Seed cotton yield		249.63**	237.4**	1808.5*	2329.0*	57.09**	60.01**	16.44	5.44	7.33
Ginning out-turn		65.164 **	51.28**	67.151*	103.5	13.489	18.16**	1.962	1.552	1.59
Hydrogen peroxide		29.950**	23.57**	326.4**	611.5**	7.286**	3.691**	22.1**	1.278	1.8 *
Catalase		2,261**	2,123**	7,143**	7,178**	598**	556**	2.0	5.0	4.0
Peroxidase		542.3**	446.5**	9,675**	90,382.*	321.5**	323.5**	54.3**	9.45*	5.32
Super-oxidase dismutase		145.4**	131.2**	38,581**	40,782.*	120.0**	87.3**	15.2	15.6*	21.0**
Total soluble protein		24.11**	18.4**	3,009**	2,978.8*	14.47**	16.6**	0.04	2.41**	2.47**
Chlorophyll contents	a	0.094**	0.10**	6.723**	8.52**	0.055**	0.04**	0.05**	0.00	0.00
	b	0.011**	0.01**	0.431**	0.59**	0.005**	0.004**	0.006**	5.01	7.94*
Carotenoids		0.011**	0.010**	4.248*	4.019**	0.005**	0.003**	0.001	0.000	0.00
Short fiber		0.900**	0.86**	0.960	1.86	1.524**	1.46**	0.074	0.02	0.079*
Fiber strength		22.62**	30.8**	29.50	3.25	13.04**	20.4**	26.18**	8.26 **	11.7**
MIC value		1.351**	1.06**	0.841**	0.26**	0.262**	0.19**	1.02**	0.10*	0.10*
Reflectance		10.44**	14.4**	225.3**	186.5*	9.09**	11.7**	0.918	2.759	2.214
Uniformity index		6.96**	22.07**	102.6*	53.5**	14.28**	16.62**	3.962	10.9**	11.0**
Upper half mean length		9.54**	11.4**	40.445	16.5	4.85**	7.6**	2.63	2.54	2.92

*Significance (α = 0.05), **highly Significant (α = 0.01).

**TABLE 3:** Genetic components of variability, genetic advance percentage means, and heritability (broad sense) estimate studied traits across normal (N) and heat stress (HT) conditions for pooled data across the years 2018 and 2019.

SOV		Max	Mini	Mean	CV%	GCV	PCV	H2b%	GAM
Plant height (cm)	N	99.80	66.00	83.68	3.86	7.33	8.29	78.29	13.37
	HT	98.80	68.20	78.49	3.45	8.49	9.16	85.85	16.20
Number of bolls	N	29.80	12.20	20.63	11.15	19.04	22.06	74.46	33.84
	HT	20.60	7.20	14.59	11.87	14.78	18.95	60.80	23.74
Boll weight (g)	N	3.75	2.31	3.01	5.46	7.18	9.02	63.32	11.77
	HT	3.74	2.04	2.92	6.36	6.77	9.29	53.17	10.17
Seed cotton yield (g)	N	69.20	29.60	50.31	5.29	15.52	16.40	89.59	30.26
	HT	69.63	26.41	40.25	8.11	22.06	23.51	88.09	42.66
Ginning out turn%	N	59.87	39.82	50.55	5.22	7.90	9.47	69.60	13.58
	HT	59.57	39.41	48.57	5.17	7.09	8.77	65.27	11.79
Hydrogen peroxide (μmol/g)	N	15.10	2.30	6.63	23.69	38.80	45.46	72.85	68.22
	HT	18.58	3.36	11.78	14.21	17.78	22.76	61.05	28.62
Catalase (U mg^–1^ protein)	N	112.00	15.00	66.54	4.82	40.36	40.65	98.59	82.56
	HT	314.00	207.10	243.21	2.24	10.06	10.30	95.26	20.22
Peroxidase (U mg^–1^ protein)	N	90.00	12.40	42.62	7.29	43.30	43.91	97.25	87.96
	HT	123.60	89.90	105.29	2.55	5.69	6.24	83.33	10.71
Superoxide dismutase (U mg^–1^ protein)	N	71.30	36.30	55.90	5.72	13.85	14.98	85.44	26.36
	HT	119.10	79.60	98.01	3.96	6.18	7.34	70.84	10.71
Carotenoids	N	0.34	0.05	0.21	12.46	26.85	29.60	82.29	50.17
(mg g^–1^ FW)	HT	0.80	0.47	0.63	6.05	8.84	10.71	68.14	15.04
Reflectance	N	84.12	69.40	75.38	3.28	3.46	4.77	52.76	5.18
	HT	76.95	68.45	72.53	1.61	2.20	2.72	64.97	3.65
The upper half mean length (mm)	N	33.14	21.92	26.89	6.82	7.40	10.07	54.09	11.22
	HT	30.01	20.92	26.04	5.44	6.50	8.48	58.81	10.28
Uniformity index %	N	97.36	80.14	86.20	3.09	3.78	4.88	59.88	6.02
	HT	87.80	80.14	84.67	2.06	2.26	3.06	54.63	3.44
Short fiber contents (%)	N	10.20	5.40	8.13	5.87	10.50	12.03	76.17	18.87
	HT	9.50	7.20	7.85	3.44	6.80	7.62	79.63	12.50
MIC (units)	N	6.90	4.00	5.12	5.25	12.01	13.11	83.94	22.67
	HT	5.90	3.80	5.23	4.06	8.50	9.42	81.43	15.80
Total soluble protein contents (mg g^–1^ FW)	N	9.42	1.18	4.65	21.92	20.17	29.79	45.86	28.15
	HT	29.33	9.30	16.03	10.85	23.85	26.20	82.85	44.71
Chlorophyll a contents (mg g^–1^ FW)	N	1.75	0.59	1.12	6.43	21.99	22.91	92.13	43.48
	HT	0.80	0.22	0.51	10.58	21.56	24.02	80.60	39.88
Chlorophyll b contents (mg g^–1^ FW)	N	0.50	0.11	0.37	7.53	20.04	21.41	87.63	38.65
	HT	0.29	0.09	0.21	12.60	20.23	23.83	72.05	35.38
Fiber strength (g/tex)	N	39.80	20.03	30.54	8.74	13.14	15.78	69.34	22.53
	HT	39.80	25.10	32.05	5.70	7.53	9.44	63.62	12.37

*SOV, Source of variation); CV%, coefficient of variation; GCV%, genotypic coefficient of variation; PCV%, phenotypic coefficient of variance); H^2^b, broad sense heritability; GAM, genetic advance per percent means.*

### Genetic Components of Various Characters Under Normal and Heat Stress Conditions

The mean values for all traits under normal and heat stress conditions were estimated. Based on these mean values following traits: PH (83.68), TNB (20.63), BW (3.01), UHML (26.89), SCY (50.31), Lint% (50.55), RD (75.38), UI (86.20), MIC (5.12), Chl-a contents (1.12), and Chl-b contents (0.37) exhibited higher mean values under normal conditions. In contrast, H_2_O_2_ (11.78), POD (105.29), TSP (16.03), SOD (98.01), Car (0.63), CAT (243.21), and STR (32.05) displayed higher mean values under high-temperature conditions (Table 2). The coefficient of variation was also computed to determine the precision of the experiment. A lower value of coefficient of variation (CV%) indicated a precise and accurate experiment. The following traits: PH, BW, Lint%, CAT, POD, SOD, SF, MIC, RD, UHML, UI, MIC, and STR had lower coefficient of variation (CV%) values under normal and stress conditions. Under both conditions, the traits TNB, SCY, Car, TSP, Chl a and b, and H_2_O_2_ had moderate to high coefficient of variation (CV%) values (Table 3). The genotypic coefficient of variation (GCV) was observed to be slightly lower than the phenotypic coefficient of variation (PCV) for all the studied traits under both conditions, indicating that the environment was the least influential on these traits. An increasing H^2^b and genetic advance mean per percent (GAM) was observed in CAT, MIC, SCY, Chl a and b contents, and PH under both conditions. Moderate H^2^b and GAM were exhibited by TNB, H_2_O_2_, Car, SF, and STR under normal and stress conditions (Table 3).

### Correlation Analysis

**FIGURE 2:**
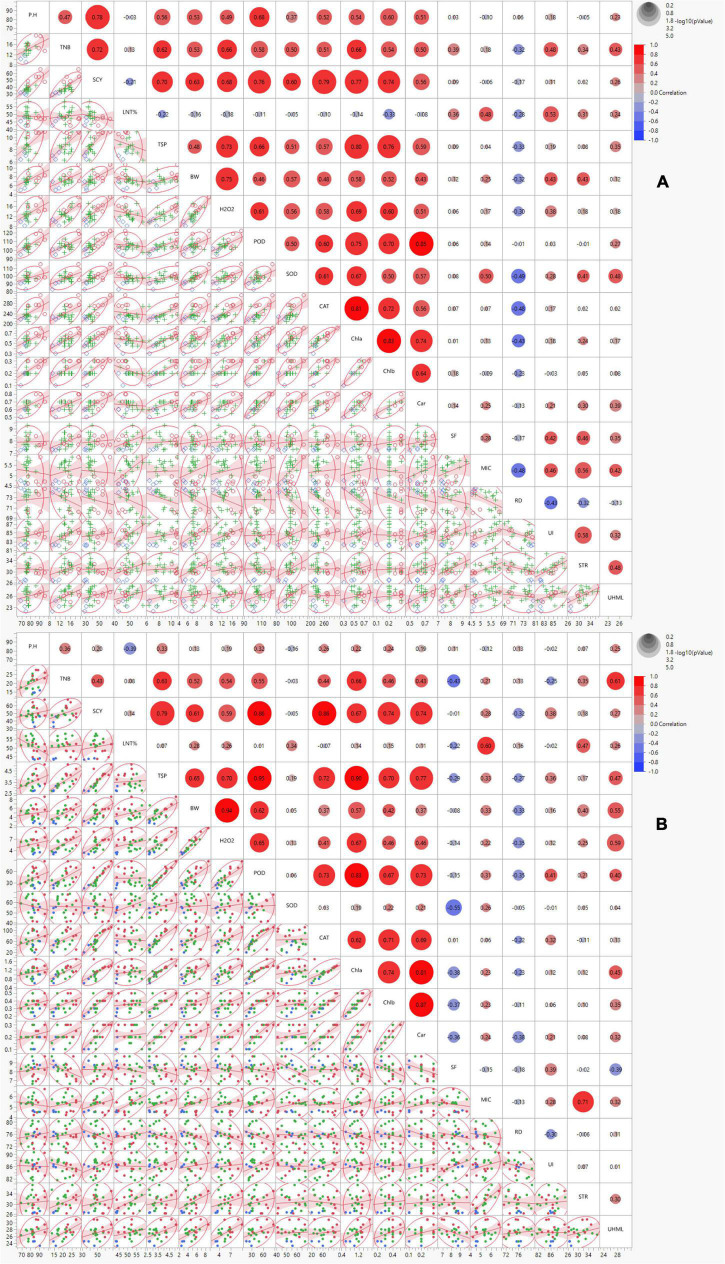
Scatterplot correlation matrix of the 19 ionic yield and fiber-related traits of 23 cotton genotypes grown under normal (left) and high-temperature stress (right) conditions. In the upper panel, red and blue circles indicated positive and negative correlations, respectively, with increasing color intensity reflecting a higher coefficient. The lower panel indicates the bivariate density distributions with ellipses between each pair of traits and trendline of the correlated traits. PH = plant height (cm), TNB = number of bolls, BW = boll weight (g), SCY = seed cotton yield (g), SF = short fiber contents (%), STR = fiber strength (g/tex), UHML = upper half mean length (mm), MIC = micronaire value (unit), RD = reflectance, UI = uniformity index (%), H_2_O_2_ = hydrogen peroxide (μmol/g), CAT = catalase (U mg^– 1^ protein), POD = peroxidase (U mg^– 1^ protein), SOD = superoxide dismutase (U mg^– 1^ protein), TSP = total soluble protein (mg g^– 1^ FW), Chl a and b = chlorophyll contents **(A,B)** (mg g^– 1^ FW), Caro = carotenoid (mg g^– 1^ FW).

Correlation analysis was performed to estimate the relationship among studied traits under normal and high-temperature stress conditions. The morphological trait, SCY, revealed significant positive associations with TSP, BW, POD, CAT, and Chl a and b under both the conditions. The biochemical trait, TSP, exhibited a significant positive association with H_2_O_2_, POD, and Chl a and b under both the conditions. BW displayed a highly significant positive association with H_2_O_2_. The biochemical traits showed a significant positive correlation with POD, CAT, Chl a and b, and Car under both conditions (Figure 2). The remainder of the correlations were inconsistently significant under both conditions, and some were insignificant or negatively correlated among themselves under normal and stress conditions. The correlation of PH with most traits in normal conditions was significantly positive, reducing TNB, SCY, TSP, BW, H_2_O_2_, POD, CAT, Chl a, and b, and Car under control stress conditions. The trait of SOD also reduced its significantly positive correlations with POD, H_2_O_2_, BW, TSP, CAT, Chl a and b, Car, MIC, STR, and UHML from normal to stress conditions. However, with SCY, TNB, PH, SF, and UI the positive correlations of SOD changed to negative ones under heat stress conditions. The negative correlation of SOD with Lint% changed to a positive correlation under heat stress. The significant positive correlation of UI with STR changed to a non-significant level under the stress condition (Figure 2).

### Cluster Analysis

Agglomerative hierarchical clustering (AHC) analysis was performed for the estimation of the degree of dissimilarity among experimental genotypes based on morphological, physiological, and biochemical traits measured under normal and high-temperature stress conditions. The cluster tree was shaped using the agglomerative hierarchical approach based on a “bottom-up” technique. The technique uses every single observation at the initial level as an individual cluster. These individual observations move forward to the next level, forming a hierarchy after pairing up successively until the final distinct cluster. The Euclidean distance method was used to calculate the distances between genotype pairs. Subsequently, all the genotypes were clustered together to create a full-fledged dendrogram *via* operating Ward’s method. A two-way clustering technique was utilized through AHC to build a two-way cluster diagram.

**FIGURE 3:**
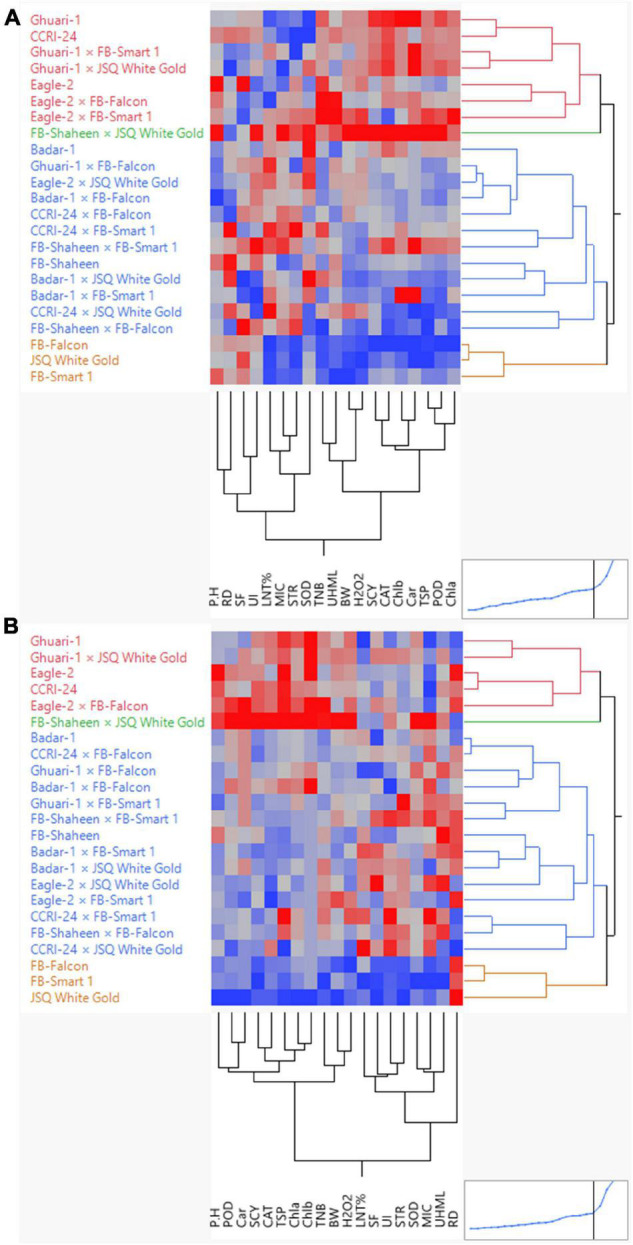
Hierarchical clustering of 23 cotton genotypes for biochemical, yield, and fiber-related traits under normal **(A)** and high-temperature stress **(B)** conditions.

This analysis divided all 23 experimental genotypes into four groups under both normal and heat stress conditions. Under normal conditions, Group-1, Group-2, Group-3, and Group-4 enclosed seven, one, 12, and three genotypes, respectively (Figure 3). Under high-temperature stress, these 23 genotypes were clustered again into Group-I, Group-II, and Group-III, comprising five, one, 14, and three genotypes, respectively (Figure 3). Different colors represented different clusters. Based on the performance of genotypes, which is depicted in the diagram through a color gradient from red to blue (highest to lowest) obtained from clustering, and under both under normal and heat stress conditions, the following genotypes performed well: FB-SHAHEEN × JSQ WHITE GOLD, CCRI-24, Ghauri-1, Eagle-2 × FB-Falcon, Ghuari-1 × JSQ White Gold, and Eagle-2 regarding agronomic, biochemical, and fiber quality attributes.

### Principal Component Analysis

**FIGURE 4:**
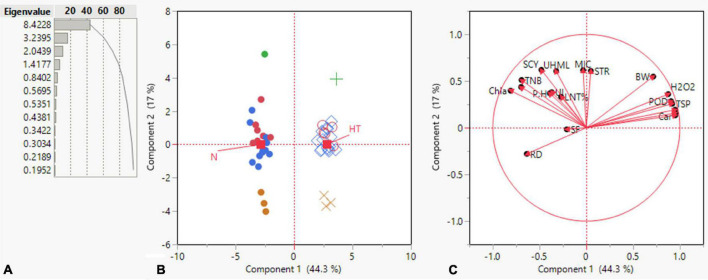
Summary of **(A)** bar chart displaying eigenvalue and variation percentage contribution by all principal components (PCs), **(B)** a biplot between PC1 and PC2 displaying the distribution of 23 cotton genotypes under normal and high-temperature stress conditions **(C)** contribution of various traits in variation for genotypes under normal and high-temperature stress conditions.

Principal component analysis is a multivariate statistical approach to studying and simplifying complicated and huge datasets. Based on the correlation among studied characters and extracted clusters, the variation patterns in cotton genotypes were also investigated using PCA to assess the genetic diversity of the genotypes and their relationship with the studied traits. Under both conditions, the total variation was divided into 19 principal components (PCs), out of which the first four PCs displayed > 1 eigenvalue. In contrast, the remaining PCs exhibited lower eigenvalues (Figure 4). The first four PCs contributed 79.56% to total variability among the cotton genotypes evaluated for various ionic, yield, and fiber quality traits under both conditions. While the remainder of all PCs shared 20.44% of the total variability under both conditions. PC-1 shared 44.3%, PC-2 exhibited 17%, PC-3 revealed 10.8%, and PC-4 displayed 7.46% of total variability among the genotypes for the studied characters. PC-1 contributed the most cumulative variability to the treatment, followed by PC-2, PC-3, and PC-4 (Figure 4).

**FIGURE 5:**
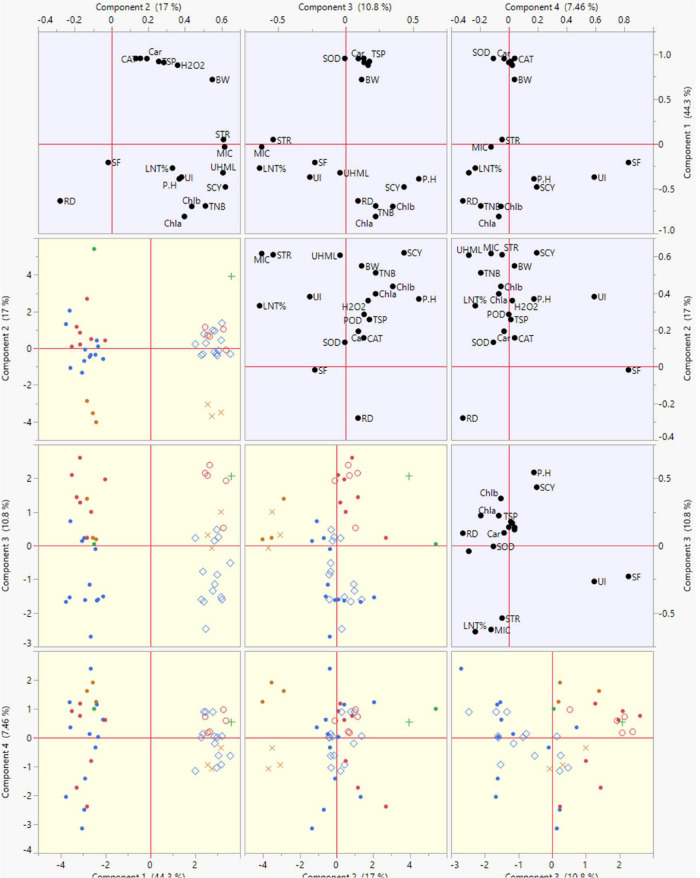
Scatterplot of PC1, PC2, PC3, and PC4 displaying their contribution to the total variability of genotypes based on studied agronomic, biochemical, and fiber-related traits under normal and high temperature stress conditions.

The summary biplot of studied traits along with their magnitudes of variation is displayed in Figure 4. All the genotypes under normal and stress conditions were distributed inside the correlation eclipse between the first two PCs (Figure 4, left). A relative distance of variables from the origin of PC-1 and PC-2 revealed a contribution of each variable to total variation for the accessions studied. It covered the plot from start to end and provided information about the diversity present among the genotypes. The second summary biplot in Figure 4 (right) between PC-1 and PC-2 explained 61.3% of the total variation. It reveals that most biochemical traits and a few others between the two PCs were positively correlated with each other: namely Car, CAT, SOD, TSP, POD, H2O2, STR, and BW. The length of vectors originating from the center is a depiction of the correlation amount among traits. These were validation of the correlations mentioned above among the studied traits under both conditions. The TNB, SCY, BW, UHML, Chl a and b, Car, H_2_O_2_, POD, TSP, and SOD had long vectors and revealed higher variation, whereas lint%, PH, MIC, STR, and UI exhibited the least variability. The SF, UI, and RD did not follow a desirable direction. PCA results displayed clear discrimination among all studied genotypes across normal and high-temperature stress conditions among the four PCs contributing to the maximum. The elaborated distribution details of studied traits under both normal and stress conditions among the four PCs are displayed in a scatterplot matrix in Figure 5. In this biplot, Car, CAT, SOD, TSP, POD, H_2_O_2_, STR, and BW show a positive correlation between PC2 and PC3 biplot, validating the correlation results.

The traits of POD, SOD, and H_2_O_2_ were closed and positively correlated in the biplot of PC-1 and PC-3. The biplot of PC-4 had the least variation compared with PC-1, with PC-2, and PC-3 being independent. In this biplot, SF and UI were more discriminating traits and had a strong positive correlation. The biplot of PC-1 with PC-4 contributed lower variation as compared with PC-2 and PC-3. In this biplot, RD, UHML, PH, and TNB lay close to each other and exhibited positive associations among themselves.

### Stress Tolerance Indices

**FIGURE 6:**
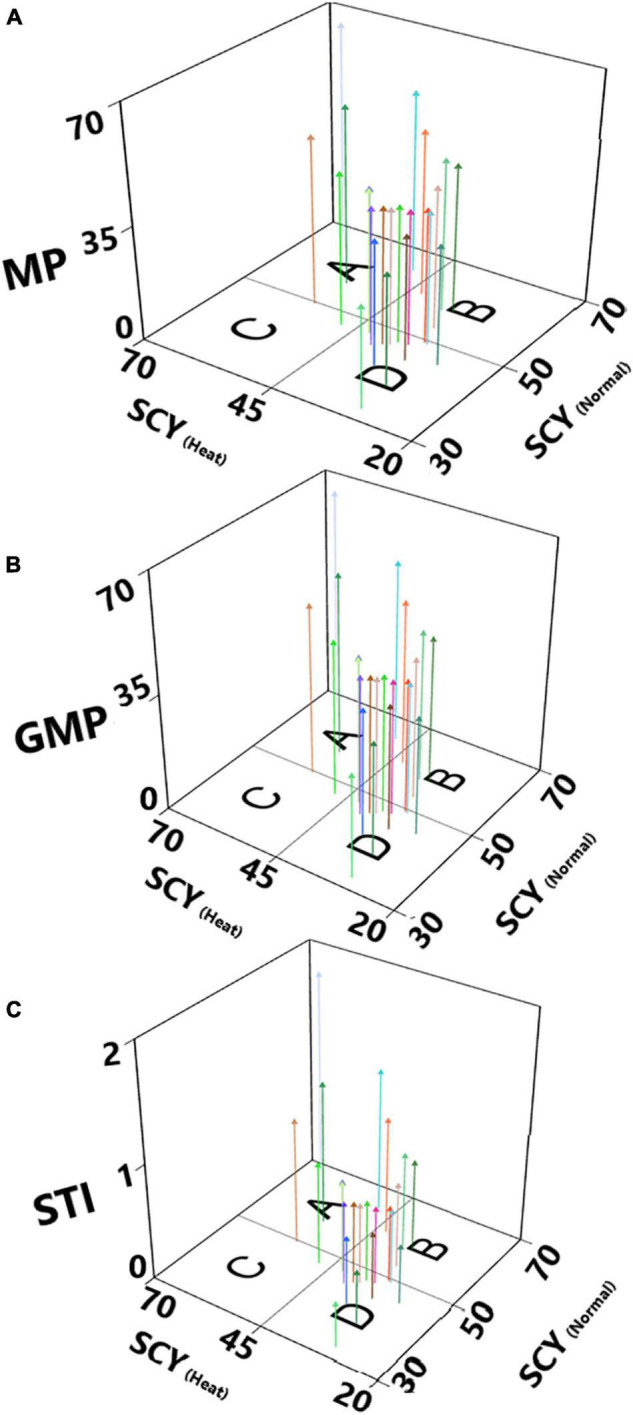
Three-dimensional scatterplots based on seed cotton yield across normal (SCY-Normal) and heat stress (SCY-Heat) conditions, and stress tolerance indices: **(A)** mean performance (MP), **(B)** Geometric mean performance (GMP) and **(C)** Stress Tolerance (STI).

We have estimated stress tolerance indices (STI) based on mean performance (MP), geometric mean performance (GMP), and STI for test genotypes, considering yield as the most critical indicator for screening regarding heat-tolerant genotypes. In this method, genotypes were ranked according to their MP, GMP, and STI. For MP and GMP genotypes were with higher values, whereas STI ≥ 1 have been considered for heat tolerance (Fernandez, 1992). Out of the 23 studied genotypes, five accessions had an STI value ≥ 1, and the highest values for STI were recorded for FB-Shaheen × JSQ White Gold (1.82), Ghuari-1 (1.27), CCRI-24 (1.26), Eagle-2 × FB-Falcon (1.11), Ghuari-1 × JSQ White Gold (1.04), and Eagle-2 (0.91) (Supplementary Table 3). According to the theory proposed by Fernandez (1992), a 3D scatterplot plot was constructed to categorize 23 test genotypes of upland cotton, including lines and their F_1_ hybrids; four groups were observed (Figure 6). The genotypes categorized as Group A had a relatively consistent performance across normal temperature and heat stress. Group B included accessions with higher performance through normal conditions; as far as group C was concerned, it comprised genotypes having high performance across the stress. In contrast, group D had genotypes with lower performance across both conditions (Supplementary Table 3). Based on cluster analysis, PCA, and STI, the genotypes FB-Shaheen × JSQ White Gold, CCRI-24, Ghuari-1, Eagle-2 × FB-Falcon, Ghuari-1 × JSQ White Gold, and Eagle-2 demonstrated superior performance under both conditions, and thus were identified as heat stress-tolerant (Figure 6 and Supplementary Table 3).

## Discussion

Of all abiotic stressors, high-temperature stress is a major constraint in improving cotton yield and production, affecting numerous attributes and physiological and metabolic processes (Snider et al., 2011; Xu et al., 2020). The development of high-yielding cotton cultivars with high-temperature resilience are needed to endure the warming global climate. To date, enormous efforts have been made to develop heat-tolerant cotton genotypes. Plant breeders’ first choice always relies on the available genetic diversity of various desirable characters among existing germplasm (Pour-Aboughadareh et al., 2018; Majeed et al., 2019). Up-to-date information regarding genetic variability and heritability is necessary to enhance breeding programs in order to develop heat-tolerant cotton cultivars (Tang et al., 1996).

Five lines and three testers were crossed in Line × Tester fashion (5 × 3), and 15 F_1_ hybrids were obtained subsequently. Using CV for the studied traits, the variation assists with enhancing crop yields by assembling beneficial genes from genetically divergent genotypes. CV also assists in depiction of the precision regarding the experiment conducted. Genetic variation is highly prone to fluctuations that take place in a plants’ environment. As the genome of a plant tries to adapt according to the vagaries of its environment, internal modifications occur to produce desirable, modified, and flexible phenotypes. Those traits exhibiting high GCV and PCV with low adverse environmental effects are advantageous for selection. Cultivars with such characters should be selected to develop desirable and adaptable genotypes (Kaleri et al., 2016; Chaudhari et al., 2017). In this work, phenotypic variance was higher than the genotypic variance for all the studied characters. The GCV was slightly lower than PCV showing the lower environmental variance, which indicates that these characters were less affected by the environment (Singh et al., 2013; Ahmadi et al., 2016). Our findings are also supported by previous reports (Khan et al., 2014).

Genetic improvement of crop plants relies on the magnitude of heritability of economic traits (Ma-Teresa et al., 1994; Ahmadi et al., 2016). Traits with high heritability and genetic advance express their features by being transmitted to the next generation in higher percentages. A high H^2^b, coupled with high GAM, may contribute to genetic gain owing to the selection process. Such a trend was observed in this work with CAT, MIC, SCY, PH, and Chl a and b content, indicating additive gene action. The mentioned traits can prove helpful in the selection of genotypes at early stages to be used further in improvement-based breeding programs. Interestingly, similar results were observed in earlier studies (Nawaz et al., 2019; Singh et al., 2019; Bhatti et al., 2020). The following traits: TNB, H_2_O_2_, Car, SF, and STR, had moderate H^2^b and GAM under both normal and stress conditions (Adhikari et al., 2018). Higher PCV, GCV, H^2^b, and GAM favor stabilized selection regarding the accumulation of alleles owing to the predominance of additive genes (Jamil et al., 2020). Some studies also suggest that heat tolerance is heritable (Snider et al., 2010, 2011).

Previous findings on high mean values are incongruent with our findings for PH (Majeed et al., 2019), Chl content (Van Der Westhuizen et al., 2020), Lint% (Azhar et al., 2009), BW (Snider et al., 2011), fiber quality traits (Snider et al., 2009), and antioxidant enzymes (Gür et al., 2010; Kamal et al., 2017; Majeed et al., 2019). In this work, as temperature elevated, H_2_O_2_ production was observed to increase; however, owing to the scavenging activity of CAT and POD, its damaging impacts were prevented (Li et al., 2007; Sekmen et al., 2014). CAT and POD convert the toxic H_2_O_2_ into water and oxygen (Farooq et al., 2018). An increase in CAT, TSP, and POD contents is generally observed in high-yielding cultivars as these are actively involved in scavenging H_2_O_2_ to maintain its optimum level (Gosavi et al., 2014; Hussain et al., 2021). The genotypes that showed higher CAT, POD, and TSP values were optimal for H_2_O_2_ level and were declared as heat-tolerant genotypes. Similar results have been observed previously in cotton (Gür et al., 2010), wheat (Sairam et al., 2000), chickpea (Kaushal et al., 2011), and moth bean (Harsh et al., 2016). Carotenoids also increase under stress conditions as they are mainly involved in safeguarding singlet oxygen (McElroy and Kopsell, 2009).

A correlation matrix is used to study the dependency of variables upon each other for better phenotypes to give improved yields (Li and Ji, 2005; Pour-Aboughadareh et al., 2021). The positively associated traits TSP, BW, POD, CAT, Chl a and b, and Car were in line with earlier reports in cotton (Wan et al., 2007). SCY exhibited a positive relationship with TSP, BW, POD, CAT, and Chl a and b content. BW showed a higher positive relationship with H_2_O_2_. Similar positive correlations among traits were also reported in earlier studies (Song et al., 2015; Majeed et al., 2019; Mangi et al., 2021).

In some cotton cultivar leaves, the antioxidant enzymes become upregulated under heat stress but remain unable to safeguard cells from oxidative injury (Snider et al., 2009). This study represented the F_1_ hybrid genotype FB-SHAHEEN × JSQ WHITE GOLD with high SCY, BW, TNB, CAT, SOD, POD, and Chl content under both normal and stress conditions. This F_1_ hybrid genotype was also observed to be superior in terms of fiber quality traits. The other F_1_ hybrids, CCRI-24 × JSQ WHITE GOLD, and EAGLE-2 × JSQ WHITE GOLD showed maximum lint%, whereas the minimum lint% was recorded for GHUARI-1 × FB-FALCON under both conditions. The parental genotypes Eagle-2 and CCRI-24 were superior in yield and fiber quality parameters under both conditions.

To select the best genotypes for agronomic, fiber-related, and biochemical traits, their discrimination from remaining low-performing ones was attained using hierarchical cluster analysis, indicating that they be utilized further in breeding programs (Chunthaburee et al., 2016). The genotypes were clustered into four distinct groups. Group-1 and Group-2 included superior performing genotypes under normal and stress conditions, discriminating them as heat tolerant. Similarly, the PCA analyses revealed the first four PCs as significant contributors to the total variation covering 79.56% toward biochemical, fiber-related, and agronomic traits. These results affirmed the differences among genotypes regarding studied traits under normal and stress conditions, which can prove helpful for their utilization in future breeding programs regarding the improvement of heat tolerance of cotton cultivars. These efficient statistical techniques are employed for the discrimination of genotypes for their diversity evaluation. The results of PCA in the current study are congruent with previous findings on cotton genotypes by other researchers (Saeed et al., 2015; Shabbir et al., 2016; Jamil et al., 2020). Out of the first four PCs the maximum contribution to the total variation residing in the experimental germplasm was from PC1 and PC2, which is in line with earlier reports related to PCA (Amna et al., 2013; Isong et al., 2017). The traits Car, CAT, SOD, TSP, POD, H2O2, STR, and BW, contributed to the first two PCs under both conditions (Javed et al., 2017). Thus, multivariate analyses are a rich source of efficiency, precision, and accuracy regarding the outcomes obtained from experimental studies.

Among the various stress tolerance indices, MP, GMP, and STI have been extensively used in various studies and are suitable selection criteria, as these parameters enable us to identify individuals with high performance regarding stress-tolerance potential under unfavorable conditions (Pour-Aboughadareh et al., 2017). In the same way, many scientists have used these indices in several crops to enable them to assess stress-tolerant genotypes for further utilization in stress breeding programs. These indices have successfully helped to discriminate the genotypes as they revealed a minimal reduction in yield in response to a stress condition, compared with the other studied genotypes. These outcomes align with the findings of other research where these indices distinguished tolerant genotypes from sensitive genotypes (Naghavi et al., 2013; Khalili et al., 2014, 2016, 2018; Etminan et al., 2019; Noorka et al., 2019). Furthermore, the grouping of genotypes for high-temperature tolerance made through these indices is almost the same as we obtained from results of hierarchical clustering and PCA, thus validating the high reliability of the methods used. Hence, tolerant accessions based on STI, AHC, and PCA results could be grown across higher temperature regions with limited penalties to their growth. The F_1_ hybrid FB-SHAHEEN × JSQ WHITE GOLD followed by Ghuari-1, CCRI-24, Eagle-2 × FB-Falcon, Ghuari-1 × JSQ White Gold, and Eagle-2 were identified as more heat tolerant as compared with the remaining experimental genotypes.

Several previous studies have documented that species with higher heat tolerance show an increasing trend in antioxidant enzyme activity in response to high-temperature stress, but susceptible species fail to do so. Thus, the evidence accumulated from current data indicates that intrinsic antioxidant resistance mechanisms of plants may exhibit a strategy for the enhancement of tolerance against heat stress. However, to perform selection efficiently for genetically transformed heat-tolerant plants, the effects of underlying mechanisms under heat stress on plant morphology, physiology, growth, and antioxidative responses must first be identified.

## Conclusion

The continuously warming global climate drives plant genotypes to adapt through the modification of specific phenotypes. With this scenario of escalating temperature, the development of cultivars that may endure abrupt fluctuations without adversely affecting yield is necessary. The first solution is to screen the available cotton germplasm for its potential against high-temperature stress. Most plants exhibit high antioxidant enzyme activities as an important step involving the heat tolerance mechanism. This work identified that the F_1_ hybrid genotype: FB-SHAHEEN × JSQ WHITE GOLD, followed by Ghuari-1, CCRI-24, Eagle-2 × FB-Falcon, Ghuari-1 × JSQ White Gold, and Eagle-2 were the best performers under stress and normal conditions as they were not adversely affected. The adverse effects of heat stress usually include disruption of routine morphological, physiological, biochemical, and fiber characters in cotton and ultimately affect yield. Potential genotypes can be efficiently employed in future cotton breeding programs to improve cotton crop yield and productivity by enhancing their heat tolerance to withstand the changing climate.

## Data Availability Statement

The original contributions presented in the study are included in the article/Supplementary Material, further inquiries can be directed to the corresponding author/s.

## Author Contributions

MZ: experimentation, data collection, and drafting the manuscript. XJ and HM: visualization, validation, review, and editing manuscript. AS: conceptualization, resources, supervision, experimentation, review, and editing. ZS: formal analysis, visualization, validation, review, and editing. AM, AI, and AR: experimentation, data acquisition, review, and editing. AA: data acquisition, experimentation, review, and editing. YY: resources, visualization, validation, review, and editing. MI: formal analysis, software, visualization, validation, review, and editing. MR: conceptualization, funding, supervision, validation, review, and editing. All authors have reviewed the manuscript critically and approved the final draft for publication in Frontiers in Plant Science.

## Conflict of Interest

The authors declare that the research was conducted in the absence of any commercial or financial relationships that could be construed as a potential conflict of interest.

## Publisher’s Note

All claims expressed in this article are solely those of the authors and do not necessarily represent those of their affiliated organizations, or those of the publisher, the editors and the reviewers. Any product that may be evaluated in this article, or claim that may be made by its manufacturer, is not guaranteed or endorsed by the publisher.

## Supplementary Material

Supplementary Table 1A Temperature recorded in the tunnel during experiments.

Supplementary Table 1B Weather data during the crop seasons of 2018 and 2019.

Supplementary Table 2 Linear mixed-effects model ANOVA for seed cotton yield of 50 cotton genotypes across normal and heat stress conditions.

Supplementary Table 3 Yield performance of 23 cotton accessions under normal and heat stress conditions, and tolerance and susceptibility indices.
